# Zebrafish and Medaka: Two Teleost Models of T-Cell and Thymic Development

**DOI:** 10.3390/ijms20174179

**Published:** 2019-08-26

**Authors:** Baubak Bajoghli, Advaita M. Dick, Annisa Claasen, Larissa Doll, Narges Aghaallaei

**Affiliations:** Department of Oncology, Hematology, Immunology and Rheumatology, University Hospital Tübingen, 72076 Tübingen, Germany

**Keywords:** T-cell development, thymopoiesis, zebrafish, medaka, imaging

## Abstract

Over the past two decades, studies have demonstrated that several features of T-cell and thymic development are conserved from teleosts to mammals. In particular, works using zebrafish (*Danio rerio*) and medaka (*Oryzias latipes*) have shed light on the cellular and molecular mechanisms underlying these biological processes. In particular, the ease of noninvasive in vivo imaging of these species enables direct visualization of all events associated with these processes, which are, in mice, technically very demanding. In this review, we focus on defining the similarities and differences between zebrafish and medaka in T-cell development and thymus organogenesis; and highlight their advantages as two complementary model systems for T-cell immunobiology and modeling of human diseases.

## 1. Introduction

The adaptive immune system is comprised of lymphocytes (B- and T-cells) and specialized organs—which provide unique microenvironments required for either the development of lymphocytes, or an effective adaptive immune response. T-cells arise from early T-cell progenitors (ETPs) originating from the hematopoietic tissue [1]. They follow a precise journey through a process called thymus homing, where they leave the hematopoietic tissue and move towards the thymus [2,3,4]. Upon entering into the thymus, ETPs follow a tightly regulated migratory pattern moving between distinct thymic microenvironments to interact with thymic epithelial cells (TECs) and other immune cell types, where they receive signal and cues required for their specification and commitment [5,6]. Developing T-cells, or thymocytes, undergo V(D)J rearrangement of T-cell receptor (TCR) genes and proceed to the divergence into either the αβ or γδ T-cell sublineage. Before exiting to the periphery as naïve T-cells, they interact with specialized TECs and dendritic cells that present tissue-restricted antigens. Recognition of self-antigens can be a death verdict for thymocytes. This process, which is called thymic selection, ensures only non-self-reactive T-cells leave the thymus [7,8].

As comprehensively reviewed elsewhere [9,10,11,12,13,14,15], the molecular and cellular mechanisms of T-cell development are evolutionarily conserved between vertebrates. Jawless fishes, which represent the most primitive living vertebrates, possess an alternative adaptive immune system based on variable lymphocyte receptors (VLRs) [16]. More specifically, it has recently been shown that they possess VLR-A^+^ and VLR-C^+^ lymphocyte sublineages, which resemble mammalian αβ and γδ T-cells, respectively [16,17,18,19,20]. Both VLR-A^+^ and VLR-C^+^ lymphocytes develop outside of the hematopoietic tissue in the thymoid, a thymus equivalent tissue located at the tip of the gill filaments [21]. The evolution of T-cell development mediated by V(D)J recombination has coincided with the emergence of gnathostomes, i.e., jawed fishes, around 420 million years ago [11]. Cartilaginous fishes, such as sharks, rays, and chimaeras are phylogenetically the oldest group of jawed vertebrates and share many molecular and cellular features of T-cell development and thymus organogenesis (so-called thymopoiesis) with mammals [10,11,12]. These similarities become apparent in teleosts [22], which are the most species-rich and diversified group of vertebrates. Among teleosts, zebrafish and medaka are two well-established model systems for T-cell research [9,11,23,24,25,26,27,28,29]. Both are small and can be easily raised side by side under the same conditions in large number and at a low cost. In this review, we focus on recent advances in our understanding of the key features of thymus organogenesis and T-cell development in these species. We continue with a description of genetic tools available for both model organisms, and then highlight zebrafish as a model system for human diseases associated with T-cells.

## 2. Thymus Development in Zebrafish and Medaka: Similarities and Differences

**Figure 1:**
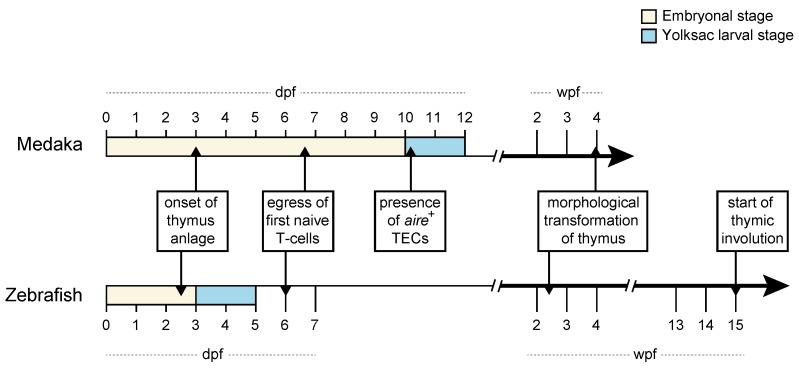
Comparative timeline of medaka and zebrafish T-cell and thymic development. The onset of thymus anlage was defined by the expression of *foxn1*, as shown previously [31,32]. The time window for the egress of first naïve T-cells into the periphery was determined using live in vivo imaging in medaka [43] and zebrafish [31]. The expression of *autoimmune regulator* (*aire*) gene, a marker for medullary TECs have been described in medaka yolksac larvae [43] but not yet in zebrafish. Note that medaka possesses a fully functional thymus with T-cells in the periphery shortly before hatching around 10 days post-fertilization (dpf) [43]. At later stages, the thymus undergoes a morphological transformation, which occurs in zebrafish between 2- and 3-wpf [39] and in medaka at 4 wpf [48]. Based on histological studies, the age-related thymic regression is prominent in zebrafish at 15 wpf [39]. In medaka, however, the starting point of thymus involution is not clear. Based on one study, the thymic regression in medaka continues until five years of age [48].

In teleosts, the thymus develops as a bilaterally paired organ, at the dorsal side of the branchial cavity, near the operculum. The time point when thymopoiesis begins in zebrafish and medaka is similar (Figure 1), as determined by the expression of *foxn1* [25,30,31,32], a transcription factor required for the differentiation of TECs [2,33]. The function of Foxn1 in defining the thymic anlage is evolutionarily conserved in jawed vertebrates [32,34]. Foxn1 directly regulates the expression of two key factors required for T-cell development, namely the *c-c chemokine ligand 25a* (*ccl25a*) and the *delta like 4b* (*dll4b*) in zebrafish and medaka [32]. The former gene is essential for the attraction of lymphoid progenitors into the thymus, and the latter gene is necessary for the specification of lymphoid progenitors into T-cells [32,35,36,37]. In contrast to mammals, TECs of teleosts also express the *foxn4* paralogue gene, albeit from the larval stage onwards [34]. Apparently, the thymic *foxn4* expression explains why *fonx1*-deficient medaka mutants only exhibit a failure of embryonic thymopoiesis and yet possess a thymus during adulthood [34]. Comparatively, mice lacking *Foxn1* do not develop thymus [2,38]. It has been, therefore, proposed that the thymic expression of *foxn4* might have been lost during the vertebrate evolution because the orthologue gene does not express in the murine thymus. This is further supported by the discovery that ectopic expression of *Foxn4* in TECs cannot compensate for the lack of *Foxn1* in the nude mice [34].

In both species, the thymus continues to grow and transforms from a small spherical shape at one week post-fertilization (wpf) to a more complex conical shape from 4 wpf onward [39]. A similar change in the shape of the thymus has also been described in sharpsnout seabream [40], indicating that this process is conserved between teleosts. Consistent with mammals, the adult thymus of teleosts has a characteristic cortical and medullary organization; however, it can range from one lobule to multiple lobules [41,42]. Zebrafish and medaka possess only one thymic lobule at each side of the body. Recently, we showed that the spatial organization of thymocytes into distinct thymic microenvironments occurs much earlier in medaka and is already distinguishable at larval stage [43]. In zebrafish, thymic compartmentalization appears only later in juveniles, between 2 and 3 wpf [39]. In both species, the thymus reaches its maximum output during adolescence, after which it undergoes age-related regression. This age-related involution of the thymus is well known in mammals [44,45]. In zebrafish, this process begins at 15 wpf [39]. However, there is considerable variation in the time point at which the thymus begins to shrink in teleosts [46]. In some teleosts, the thymic structure can even remain intact for the entire life [47]. This is apparently true for medaka because histological analysis showed that the thymus of a three years old fish was similar to that in three-month-old fish [48]. Given that the rapid decline in thymus size is considered to be a detrimental process [45], a comparative analysis between zebrafish and medaka would offer substantial advantages for studying the molecular mechanisms of age-associated thymic involution.

## 3. T-Cell Development in Zebrafish and Medaka

**Figure 2:**
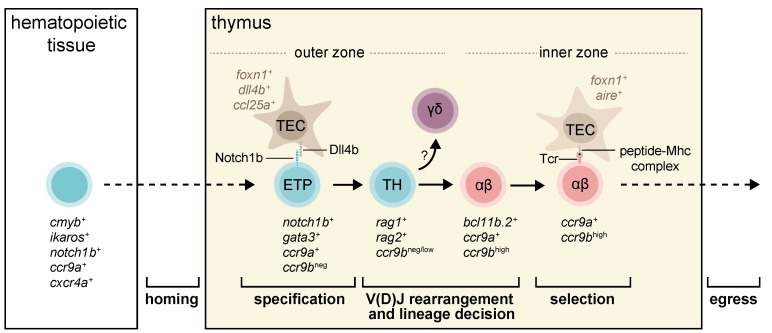
The current model of T-cell development in teleosts. A schematic illustration of stages of T-cell development in zebrafish and medaka, highlighting the expression of marker genes at each developmental stage based on previous studies [31,32,43]. Dashed arrows mean migration into and out of the thymus. Arrows mean development into a next stage. Abbreviations: ETP, early T-cell progenitor; TEC, thymic epithelial cells; TH, thymocytes.

In this section, we separated the process of T-cell development into four broad steps (Figure 2). In the first step, lymphoid progenitors colonize the thymus; the second spans T-cell commitment and the divergence of αβ and γδ T-cells lineages. The third step is the selection of functional and non-self-reactive T-cells. Lastly, mature T-cells exit the thymus into the periphery. At each step, we highlight the major gaps in our current knowledge of zebrafish and medaka.

### 3.1. The Entry of Lymphoid Progenitors into the Thymus

**Figure 3:**
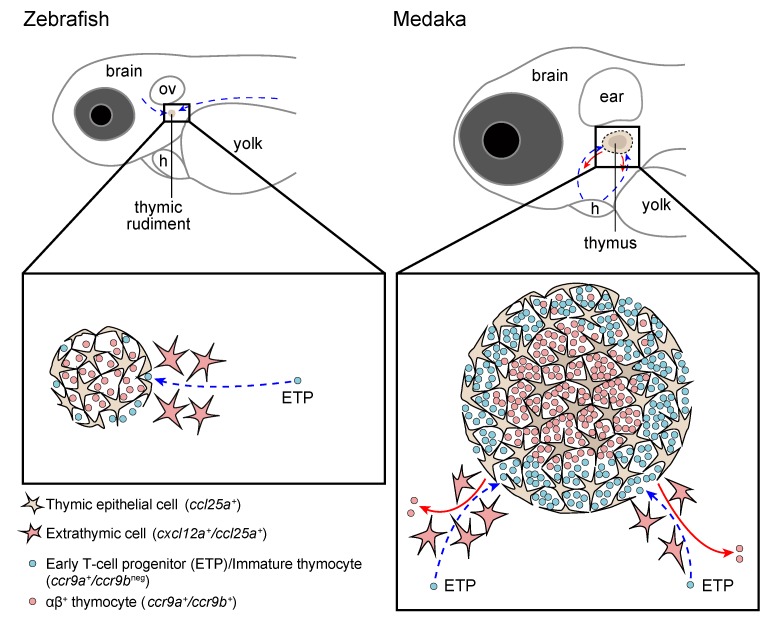
Early colonization of the thymus by lymphoid progenitors in freshly hatched zebrafish and medaka larvae. A schematic illustration of the migratory paths of thymus colonization in zebrafish (left panel) and medaka (right panel) at the time when they hatch out of the chorion according to previous studies [30,31,42,48]. Blue dashed arrows indicate the migration path of cells from the extrathymic mesenchyme into the thymus. Red arrows indicate the migratory pathways of RTEs into the periphery. Abbreviations: h, heart; ov, otic vesicle.

The seeding of the thymus with lymphoid progenitors can be mediated either by vasculature-dependent or -independent pathways [5]. In zebrafish and medaka, the thymus at embryonic and larval stages is not vascularized, and the blood circulation plays a subordinate role in this process [31]. Long-term in vivo imaging of transgenic zebrafish embryos showed that lymphoid progenitors use defined ways to migrate through the mesenchyme into the embryonic thymus (see Figure 3 and for more information Ref. [31,32,43,49]). The migration of ETPs from the mesenchyme into the thymus resembles the colonization of the mouse thymus before vascularization, which occur at E15–16. Our results highlighted, for the first time, that a combination of chemokines are responsible for the attraction of ETPs into the thymus [32]. We showed that knockdown of two chemokines, *ccl25a* and *cxcl12a*, impairs the process of thymus homing in zebrafish and medaka [32]. We, along with others, have shown that TECs and stromal cells in the thymic periphery mainly express the former chemokine, while the latter chemokine is expressed in stromal cells surrounding the thymus. ETPs via expression of *c-c chemokine receptors 9a* (*ccr9a*) and *c-x-c chemokine receptors 4a (cxcr4a*) respond to this chemotactic milieu and enter into the thymus [31,32,43]. This molecular mechanism is evolutionarily conserved between fish and mammals. In mice, ETPs are unable to colonize the thymus when they lack chemokine receptor *Ccr9*, *Cxcr4* and *Ccr7* [50]. It is worth noting that the latter chemokine receptor does not express in the medaka thymus at the larval stage.

### 3.2. Commitment of Thymocytes

Lymphoid progenitors are pluripotent cells and their interaction with the thymic environment, which provides essential developmental signals and cues, is essential for their proliferation and commitment to T-cell lineages. One of the crucial steps in establishing T-cell identity is the activation of the Notch signaling pathway [37,51,52,53]. In particular, Notch1 receptor on the surface of lymphoid progenitors interacts with its non-redundant ligand Dll4 expressed by TECs, which leads to activation of gene regulatory networks involved in T-cell specification. Although T-cell specification has been extensively studied in mice [54,55,56], the underlying mechanisms in teleosts are poorly understood.

Both Notch1 and Dll4 genes are duplicated in the genomes of zebrafish and medaka [32]. Functional analysis suggested that lack of zebrafish *notch1a* and *notch1b* impairs the development of hematopoietic stem cells [57], making a more detailed understanding of their possible functions during thymic T-cell development highly desirable. One way to address the role of Notch signaling in T-cell development was knockdown of medaka *dll4b*, which is expressed by TECs [32]. This study has shown that *dll4b* is downstream of Foxn1 and is required for the expression of the *T-cell receptor β gene* (*tcrb*). As mammalian Notch signaling also contributes to αβ or γδ T-cell decision [54], it remains unclear whether the lack of Notch signaling would also affect the choice between T-cell sublineages in teleosts.

Overall, there is not much known about the mechanisms controlling the choice between αβ and γδ sublineages in teleosts. In mice, the formation of pre-TCR, a complex that is composed of a TCRβ chain plus an invariant of the pre-TCRα chain, is a critical step in lineage decision [54,58]. Interestingly the gene encoding for pre-TCRα chain (*PTCRA*) is present only in the genomes of mammals, some reptiles and birds [59]. Lack of this gene in the genomes of teleosts would argue that the mechanism of lineage decision might be different in lower vertebrates. Further work is needed to clarify this issue.

### 3.3. Selection of Functional and Non-self-reactive Thymocytes

After completing differentiation, thymocytes must pass positive and negative selections, which are critical checkpoints governing the development of functional and self-tolerant T-cells. The first checkpoint verifies proper TCR protein expression in thymocytes because most V(D)J rearrangements mediated by *recombination activating gene* (*Rag*) are out-of-frame and do not give rise to genes encoding a functional protein [60]. In the second checkpoint, thymocytes are exposed to tissue-specific antigens either presented by dendritic cells or induced by the transcription factor *Aire* in medullary TECs [61,62,63]. Thymocytes carrying TCRs with the highest avidity for self-antigens undergo negative selection. Very little is known about these processes in zebrafish, presumably because studies on T-cell development are mostly limited to five days post-fertilization (dpf), and, at this stage, the thymic medullary region has not yet developed. Furthermore, suitable zebrafish transgenic fluorescent-based reporters that enable direct monitoring of the thymic selection event are not yet generated. By using a medaka *aire*::*gfp* knock-in reporter line, we recently detected a patch of *aire*-expressing cells located in the dorsocentral region of the larval thymus at 10–12 dpf [43]. αβ thymocytes located in this thymic region are characterized by the expression of chemokine receptor *ccr9b* [43]. Time-lapse in vivo imaging revealed a dynamic interaction between *ccr9b*-expressing thymocytes and dendritic cells. The latter cell type has the ability to interact, engulf and phagocyte thymocytes during the negative selection [43]. Given that thymocytes express various chemokine receptors to control their migratory behavior or positioning within the thymus, it is still unclear whether *ccr9b* is required for the positioning of thymocytes within the thymus or if it is only involved in the process of thymic selection.

### 3.4. Egress of Thymocytes

The emigration of thymocytes from the thymus into the periphery is an active process controlled by signals mediated by *sphingosine-1-phosphate receptor 1* (*S1PR1*), which belongs to the G-protein-coupled receptor family [64]. In zebrafish, the role of the *s1pr1* gene was investigated mainly in vascular development [65,66,67,68], and nothing is known about its possible function in thymic egress.

Recently, a photoconvertible reporter fish was used to determine when the first T-cells emigrate from the zebrafish thymus [31]. In this study, thymocytes were photoconverted at the onset of intrathymic T-cell development, and their appearance outside the thymus was monitored. Based on this experimental setup, T-cells first arrived at the kidney at 6 dpf. However, it is not clear if the kidney is the organ that thymocytes preferentially colonize after leaving the thymus. In medaka, the T-cells that first left the thymus were found in the intestine and perivascular space in the trunk region around 6–7 dpf [43]. Our observations also suggested that recent thymic emigrants (RTEs) do not enter into the primary head sinus, which is the main vein collecting the blood bilaterally from the head and is the next closest vein to the thymus. Live in vivo imaging showed that RTEs preferentially use the same migratory paths that had been used to colonize the thymus [43], as illustrated in the bottom panel of Figure 3.

Finally, it is worth noting that the emigration of first T-cells from the thymus occurs at the post-hatching stage in zebrafish, whereas medaka embryos shortly before hatching have a fully functional thymus with T-cells in the periphery, as illustrated in Figure 1 and Figure 3. A possible explanation is that zebrafish embryos hatch out of the chorion much earlier than medaka.

## 4. Genetic Tools to Study T-cell Development in Zebrafish and Medaka

Zebrafish and medaka provide many advantages for T-cell research. Experimental manipulations can efficiently be conducted in both species with slight modifications. For instance, application of a heat-inducible promoter [69] enables the temporary induction of a gene-of-interest. This approach has been used to examine the functions of transcription factors Foxn1 and Bcl11b, as well as the chemokine Ccl25a during T-cell development [32,70]. To perform loss-of-function analysis, the morpholino-based knockdown experiment is one classical method of choice. An advantage of this approach is the rapid generation of knockdown phenotypes in wild-type embryos without the need for classical mutagenesis screens or the development of mutants. One caveat of this method, however, is that the induced phenotype is often more severe than that of the corresponding mutants. Apart from this approach, the main tool-of-trade for biologists working with zebrafish is a cadre of mutants that were generated using large-scale random mutagenesis [71,72]. Long-term characterization of zebrafish mutants that displayed reduced thymocytes within the embryonic thymus resulted in the identification of a range of T-cell development regulators. As proof of concept, many mutations were identified in genes, such as *rag1*, *cmyb*, *jak1*, *jak3*, and *interleukin-7 receptor*, which are known to be involved in mammalian T-cell development [73,74,75,76], emphasizing the evolutionary conservation of this process in vertebrates. Apart from that, screening of zebrafish randomized mutations surprisingly revealed that genes essential for DNA replication, DNA repair or processing of pre-mRNA specifically affect T-cell development [77,78,79]. A similar random mutagenesis screen was also conducted in medaka [25]. Characterization of one of the generated medaka mutants resulted in the identification of *WDR55*, a nucleolar modulator of ribosomal RNA biosynthesis, which is involved in the development of the thymic primordium [80]. In another study, two medaka mutants carrying nonsense and frameshift mutations in the *foxn1* gene were used to study the function of *foxn1* in thymopoiesis [34]. In the context of loss-of-function analysis, there is currently an increasing tendency to generate gene knockout lines by CRISPR-Cas9 mediated gene-editing techniques [81,82,83]. Moreover, recently improved protocols enable efficient knock-in in both species [84,85].

Apart from their strength as genetic model systems, the most crucial advantage of zebrafish and medaka is their optical transparency. Furthermore, the thymus is located superficially, close to the skin, which provides better access for imaging. As reviewed recently [86], both zebrafish and medaka have distinct benefits, but each comes with certain caveats. The small body size of zebrafish larvae permits to monitor the migration of lymphoid progenitors from the hematopoietic tissue towards the thymus. However, the zebrafish thymus at this stage is populated only by 20–50 thymocytes [31]. This limitation can be circumvented by using freshly hatched medaka larvae, where the thymus contains more than 1000 thymocytes [43]. The capability of noninvasive live imaging provides the benefit to study dynamic processes associated with T-cell development, such as thymus homing, intrathymic cell trafficking, thymic selection, and emigration of thymocytes into the periphery in a quantitative manner. Thus far, various transgenic fluorescence-based reporter lines have been developed in both species to monitor TECs [31], lymphoid progenitors [32,43], thymocyte subsets [24,43], naïve T-cell [26,43], as well as regulatory T-cells [87]. Taking advantage of the transgenic reporters combined with functional analysis enables researchers to directly address the role of a gene-of-interest during thymic T-cell development.

Perhaps the most technical limitation in zebrafish and medaka is the lack of monoclonal antibodies for T-cell surface markers, which enable better characterization of developmental stages or distinguish various subtypes of T-cells. This limitation can be partially circumvented by using transgenic fluorescence-based reporter lines. One caveat is that most transgenic reporters carry a construct in which the expression of a reporter gene is under the control of the proximal promoter of a cell-specific gene, and lack possible distal regulatory regions. For example, the transgenic construct of the *lck:gfp* line includes only the proximal promoter [26,43], which is transcriptionally active in thymocytes but not in all T-cell lineages [88]. Thus, only thymocytes and RTEs can be visualized by using this reporter line. However, this caveat is less of an issue when the reporter gene is directly integrated into a gene locus using the CRISPR-Cas9 technique. Undoubtedly, investments in the development of monoclonal antibodies against various surface markers will push forward the exploration of T-cell biology in zebrafish or medaka to the next level.

## 5. Zebrafish as Model System for T-Cell Diseases

Zebrafish are being used as a powerful model system for studying human diseases, such as T-cell acute lymphoblastic leukemia (T-ALL), T-lymphoblastic lymphoma (T-LBL), which are malignancies caused by the clonal expansion of malignant thymocytes [89,90]. One of the zebrafish T-ALL models is driven by transgenic MYC under the control of the *rag2* promoter [91,92,93]. This zebrafish model was used to identify the role of tumor suppressor gene PTEN [92] and the proapoptotic protein BIM in a Myc-induced T-ALL model [94]. As the *rag2* promoter is transcriptionally active during the development of T-,cells as well as B-cells, it has recently been shown that B-cell acute lymphoblastic leukemia (B-ALL) could also occur in this zebrafish T-ALL model [95]. Thus far, various zebrafish T-ALL models have been developed based on the constitutive expression of the human intracellular domain of *NOTCH1*, mouse *Myc* [91], mouse *Akt2* [92], zebrafish *jdp2* [96] and human *ARID5B* [97] genes. Besides these transgenic zebrafish T-ALL models, Frazer and colleagues identified three zebrafish mutants, *Hulk* (*hlk*), *Shrek* (*srk*) and *Oscar the grouch* (*otg*) that develop transplantable T-ALL using a forward genetic approach [98].

Recently, zebrafish has been used as a model for severe combined immunodeficiency (SCID), which is a congenital disorder characterized by a significantly low number or defective function of T- and B-cells [70]. In this work, the effect of a mutation in the BCL11B gene that was discovered in a SCID patient was tested on T-cell development using zebrafish. Punwani and colleagues used the heat-inducible system [69] to express the human *BCL11B* mutant in zebrafish embryos ectopically, and the results mimicked the patient’s phenotype [70]. They showed that knockdown of zebrafish *bcl11b* impairs T-cell development, which supports our previous study [32]. Another study has shown that *zap70* mutant zebrafish exhibit reduced numbers of T-cells [99], which resembles SCID patients who have ZAP70 deficiency. A group of patients with atypical SCID, also known as Omenn syndrome, harbor missense mutations in the *RAG1* or *RAG2* genes leading to a partial impairment of V(D)J recombination [100]. Similarly, a lack of mature T-cells but a reduced B-cell repertoire were observed in a zebrafish *rag2* mutant, which produces a protein that is truncated after amino acids 450 of the 503 amino acids long Rag2 protein [101]. Overall, zebrafish has proven to be a powerful model system for modeling human diseases associated with T-cell development.

## 6. Conclusions

Over the past decade works from many laboratories, including our own, have established zebrafish and medaka as two invaluable model systems for studying T-cell development and thymus organogenesis. In recent years, novel methods and technologies have been implemented to shed light on the mechanisms controlling thymus homing, intrathymic cell trafficking, and to identify previously unknown gene regulators of T-cell development. These works have demonstrated that zebrafish and medaka have the potential to be used to address unanswered questions, which are, in mice, very technically demanding [86]. Despite substantial recent progress, several questions remain unanswered or poorly understood (Box 1). For example, the full diversity of T-cell subtypes is not yet characterized in zebrafish and medaka. A comprehensive understanding of T-cell subtypes in these two species will allow further study of their ontogeny and functional plasticity upon environmental changes. In this context, the use of single-cell RNA sequencing will provide not only a wide picture of T-cell diversity but also help identify new cell type-specific markers that can be later used to generate fluorescent-based reporter lines. Considering that medaka and zebrafish lineages separated about 314–332 Myr ago [102], studies in both species will provide complementary information, and can be integrated to maximize insight into the general principles of vertebrate T-cell development and thymopoiesis.

Box 1 **Open questions in the field of thymic and T-cell development in teleosts.**

- To what extent are the underlying mechanisms of T-cell commitment and exit into the periphery conserved between teleosts and mammals?
- How is the choice between αβ and γδ T-cell sublineages regulated in teleosts?
- How does the morphological transformation of the thymus in adolescent fish change the cellular composition and spatial gene expression patterns within the thymus?
- Why does age-related thymus involution not occur in some teleost species?
- In mammals, naïve T-cells can differentiate into various subtypes, including cytotoxic T-cells, helper T-cells (e.g., Th1, Th2, Th17), and regulatory T-cells (Treg). What is the full diversity of T-cell subtypes in teleosts?

## Abbreviations

AIRE	Autoimmune regulator
dpf	Days post-fertilization
ETP	Early T-cell progenitor
RTE	Recent thymic emigrants
T-ALL	T-cell acute lymphoblastic leukemia
TCR	T-cell receptor
TEC	Thymic epithelial cell
T-LBL	T-lymphoblastic lymphoma
VLR	Variable lymphocyte receptor
wpf	Weeks post-fertilization
